# 5^th^ International AIDS Society Conference on HIV Pathogenesis, Treatment and Prevention: summary of key research and implications for policy and practice – Operations research

**DOI:** 10.1186/1758-2652-13-S1-S5

**Published:** 2010-06-01

**Authors:** Rodney Kort

**Affiliations:** 1 Kort Consulting, Toronto, M4Y 2T6, Canada

## Abstract

Operations research was added as a fourth scientific track to the pathogenesis conference series at the 5^th^ IAS Conference on HIV Pathogenesis, Treatment and Prevention (IAS 2009) in recognition of the importance of this growing research field and the need for applied research to inform and evaluate the scale up of some key interventions in HIV treatment, care and prevention.

Several studies demonstrated how task shifting and the decentralization of health services can leverage scarce health care resources to support scale-up efforts. For example, a Ugandan study comparing home-based and facility-based antiretroviral therapy (ART) delivery found that both delivered equivalent clinical outcomes, but home-based delivery resulted in substantial cost savings to patients; and a retrospective cohort analysis of an HIV care programme in Lesotho demonstrated that devolving routine patient management to nurses and trained counsellors resulted in impressive gains in annual enrolment, retention in care and other clinical indicators.

Studies also demonstrated how the use of trained counsellors and public health advisors could effectively expand both clinical and public health capacity in low-income settings. Studies evaluating the impact of integrating HIV and TB care resulted in improved treatment outcomes in coinfected populations, the development of environmental interventions to reduce TB transmission, and uncovering of the extent of multi-drug-resistant and extremely drug-resistant tuberculosis (MDR-TB and XDR-TB) in KwaZulu-Natal, South Africa.

Some mathematical modelling and cost-effectiveness studies presented at this meeting addressed interventions to increase retention in care, and strengthened the evidentiary basis for universal voluntary testing and immediate ART on reducing HIV transmission; debate continued about the relative merits of clinical versus laboratory monitoring. Finally, a provocative plenary presentation outlined the shortfalls of current prevention interventions and argued for more cost-effectiveness analyses to guide the selection of interventions for maximum benefit.

## Discussion

The new operations research track at IAS 2009 reflects a growing recognition of the need for applied research to inform the scale up of HIV programmes. The wide range and quality of presentations from a variety of disciplines exemplified one of the challenges for the conference: how to clearly define the operations research field in order to improve understanding of its role among investigators and other HIV professionals. In his summary report, Track D lead rapporteur George Schmid (World Health Organization, Geneva) defined operations research as research that “provides decision makers with information to improve the performance of their programmes ... including identifying service delivery problems and evaluating a variety of delivery interventions” [1].

Key research presented in Track D included: programme evaluations; evaluations of various service delivery models; epidemiology; mathematical modelling; and cost-effectiveness studies. A pre-conference workshop convened by the International AIDS Society (IAS) provided concrete information on how to use operations research methodologies to strengthen HIV prevention, care and treatment scale up. The need for new operations research to further build the evidence base for the health systems strengthening effects of HIV investments also dominated discussion at a two-day pre-conference meeting convened by the IAS.

### Strategies for strengthening service delivery

Several studies highlighted how task shifting and the decentralization of HIV services can leverage scarce health care resources to support scale-up efforts. Shabbar Jaffar (London School of Hygiene and Tropical Medicine) compared traditional facility-based delivery of ART with home-based care delivered by trained lay people (including medication provision, adherence support and referrals) in Uganda, and found that both resulted in excellent and equivalent clinical outcomes based on mortality, CD4+ count and virologic response. While the cost of service provision was similar for both models, the home-based care intervention resulted in substantially reduced costs for patients [2]. 

Lipontso Makakole (Scott Hospital, Morija, Lesotho) presented encouraging results from a retrospective cohort analysis of a country programme that devolved routine patient management to nurses and referred patients coinfected with HIV and TB to specially trained counsellors. After two years, annual enrolment more than doubled, the proportion of adults presenting with less than 50 CD4 cells/mm^3^ was reduced from 27% to 13%, and 80% of patients were retained in care [3].

Other presenters offered additional examples of strategies to expand health system capacity. In Rwanda, Alphonse Kayinanga (Catholic Relief Services/AIDS Relief) and colleagues documented a programme that trained community volunteers to screen 3340 HIV-positive individuals for TB, successfully referring 400 for clinical assessment [4]. Pius Tih (Baptist Convention Health Board, Cameroon) described a public health programme in Cameroon that used trained health advisors to conduct contact tracing and partner notification for individuals testing positive in a high-prevalence region of the country [5].

Operations research also demonstrated how to better integrate service delivery among HIV programmes and among HIV and non-HIV health services. In his plenary presentation, Gerald Friedland (Yale School of Medicine, New Haven) described how an integrated HIV and TB treatment programme in KwaZulu-Natal, South Africa – where more than 90% of TB cases are coinfected with HIV – not only resulted in substantially better clinical outcomes, but also uncovered the extent of multi-drug- and extremely drug-resistent TB prevalence and their effect on morbidity and mortality [6]. Friedland underscored a consistent theme: combinations of activities and approaches (e.g., using a variety of interventions to reduce clinical and environmental factors that contribute to the spread of TB), rather than a single approach, are likely to yield the most benefit.

The importance of using a combination of approaches to deliver HIV and related health services was echoed in other Track D studies on service integration, including a study of Family Health International programmes in five African countries that assessed the extent to which reproductive health services were integrated into counselling and testing, and care and treatment programmes. The results suggest that, beyond condom provision, more work needs to be done to better integrate HIV with sexual and reproductive health services for women [7].

Not all task-shifting interventions are created equal, however, as demonstrated by a study presented by Charity Kabondo (University of North Carolina Project, Lilongwe, Malawi) where trained traditional birth attendants (used by 44% of pregnant women) were used to expand the delivery of antiretroviral (ARV) prophylaxis to prevent vertical transmission. Although the use of traditional birth attendants expanded the use of ARV prophylaxis beyond traditional facility-based care, fewer than half of the pregnant women were identified as HIV positive and less than one-quarter of infants diagnosed as HIV positive received nevirapine prophylaxis from traditional birth attendants [8].

### Expanding ART access and retention

Yves Souteyrand (WHO, Geneva) presented encouraging data on increases in ART coverage in 2008, including an almost 50% one-year increase in coverage among children in sub-Saharan Africa [9]. Global ART coverage is currently estimated at 35% (45% for children under 15 years of age). However, evidence from a number of operations and clinical research studies demonstrates that earlier ART initiation for individuals with CD4+ counts under 350 cells/mm^3^ results in substantial reductions in morbidity and mortality. Such evidence is expected to change World Health Organization normative guidelines (released in November 2009), thereby making many more people eligible for treatment and reducing treatment coverage figures [10].

Against this backdrop, several studies addressed the vexing problem of ART programme retention. Kenneth Freedberg (Massachusetts General Hospital, Boston) presented a modelling and cost-effectiveness study of four lost-to-follow-up interventions (elimination of ART co-payments, provision of free opportunistic infection medications, increased health care worker training and coverage of transportation costs), which indicated that such interventions would be highly cost effective [11]. Based on five years of operations research, Anthony Harries from the IeDEA Collaboration identified 10 steps to improve access and retention, ranging from simple monitoring and evaluation activities to creative reward programmes for overstretched health care workers [12].

In an excellent overview of male circumcision delivery in sub-Saharan Africa, Agot Kawango emphasized that while most countries have completed the necessary situation analyses and taken steps to develop political support and community messaging for male circumcision roll out, health care worker shortages and health facility preparedness remain significant barriers to scale up.

Encouragingly, she noted a number of operations research studies underway aimed at evaluating several issues related to roll out, including safety, impact on sexual risk behaviours, HIV incidence and the comparisons of different intervention models, such as physician versus non-physician delivery. One study on male circumcision implementation in Uganda, included in Kawango’s overview, indicated no significant difference in adverse event rates by technique, health facility or cadre of health care worker [13].

In other HIV prevention-related operations research, a promising modelling study on universal voluntary testing and treatment, presented by Reuben Granich (WHO, Geneva), demonstrated the potential preventive effects of this strategy at a population level [14]. Questions remain on how this population-based approach could be implemented.

### The monitoring debate: the role of laboratory services

**Figure 1:**
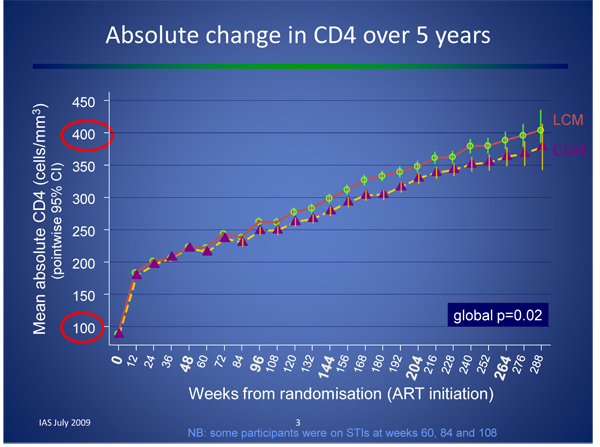
**Laboratory (LCM) versus clinical (CDM) monitoring in the DART study** Laboratory (LCM) versus clinical (CDM) monitoring in the DART study Source: Mugyenyi P, et al: **Impact of routine laboratory monitoring over 5 years after antiretroviral therapy (ART) initiation on clinical disease progression of HIV-infected African adults: the DART Trial final results**. 5^th^ IAS Conference on Pathogenesis, Treatment and Prevention. TUSS103. [15]

While there is consensus regarding the need for a baseline CD4+ count to guide initial clinical decisions, the frequency of subsequent CD4+ counts and the use of viral load monitoring was the subject of substantial debate at IAS 2009. Peter Mugyenyi (Joint Clinical Research Centre, Kampala) reported on final results from the five-year Development of Anti-Retroviral Therapy in Africa (DART) study, which compared clinical to laboratory monitoring using progression to WHO Stage 4 event or death as endpoints. There was no statistically significant difference between the two arms within the first two years and a small, but statistically significant difference from year three onwards, explained by slightly later switch rates in the clinical monitoring arm. Nevertheless, survival rate was excellent in both arms, at 87% and 90% for clinical and laboratory monitoring, respectively (see Figure 1) [15].

A cost-benefit analysis of the DART results indicated that laboratory monitoring for toxicity was expensive and provided no significant benefit, and that CD4+ monitoring may be cost effective as a targeted (rather than routine) strategy [16,17]. A modelling study presented by Sylvia Ojoo (University of Maryland/Institute for Human Virology, Nairobi) also supported these data, suggesting that CD4+ count monitoring at six, 36 and 60 months was nearly as clinically effective as monitoring every six months and substantially more cost effective [18].

### Financing and cost effectiveness: more and better

**Figure 2:**
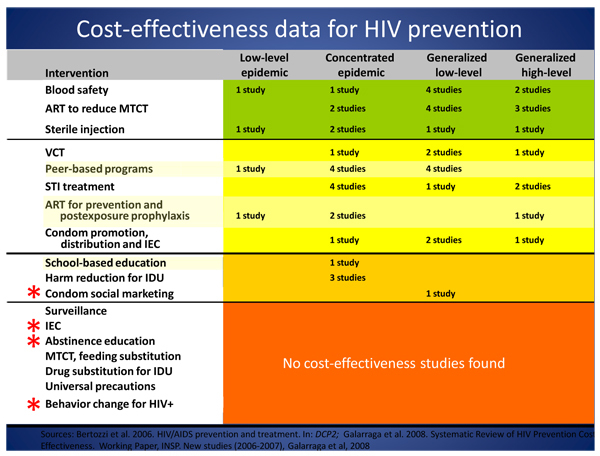
**Cost-effectiveness data for HIV prevention** Sources: Bertozzi S, et al: **HIV/AIDS prevention and treatment**. In: *DCP2;* Galarraga et al: *Systematic Review of HIV Prevention Cost Effectiveness*, 2008. Working Paper, INSP. New studies (2006-2007), Galarraga et al, 2008

A number of speakers referenced concerns regarding the potential impact of the global economic recession on HIV commitments from both government and private donors. In a provocative plenary presentation, Stefano Bertozzi (Instituto Nacional de Salud Publica, Mexico City) argued that while the HIV field has been successful in securing more money for AIDS in recent years, it has been less successful in getting “less AIDS for the money”. He pointed out that few cost-effectiveness studies have been conducted on many common prevention interventions (see Figure 2), and that too often, interventions are not chosen strategically or used where they will have maximum benefit [19]. Bertozzi called for strategies to improve the efficacy and cost effectiveness of current interventions, while advocating for additional investments to close the more than US$8 billion funding gap.

Paul Sax (Brigham and Women’s Hospital/Harvard Medical School, Boston) presented the results of a cost-effectiveness analysis of ACTG 5164, a trial in which patients with opportunistic infections were randomized to receive either early or deferred ART [20]. While the results are not necessarily generalizable to resource-limited settings, the finding is consistent with other studies demonstrating better clinical outcomes and reduced mortality when ART is initiated for everyone with less than 350 CD4+ cells/mm3, including the landmark Comprehensive International Program of Research on AIDS (CIPRA) HT001 study conducted in Haiti. The Data Safety and Monitoring Board recently suspended CIPRA HT001 based on the significant interim differences between the deferred and early ART initiation arms [21].

Marielle Bemelmans (Médecins Sans Frontières, Brussels, Thyolo) presented a landmark cost-effectiveness study of the Médecins Sans Frontières-Ministry of Health ART programme in Malawi, demonstrating that a variety of measures, including task shifting, decentralization of care to health centres and community involvement, helped that country meet universal access targets (with 78% programme retention by the end of 2007) while keeping the marginal cost of the ART programme to €2.6 per inhabitant per year due to economies of scale [22].

## Conclusions

There is some evidence that new operations research studies presented at IAS 2009 had an immediate impact on some policy makers in the conference’s host country; an article published after the conference noted that the South African National AIDS Council would be meeting to discuss issuing guidance for earlier ART initiation, treatment for all infants diagnosed with HIV, and better integration of TB and HIV programmes [22]. The evidence referenced earlier in this article, which demonstrates how innovative service delivery models can successfully leverage community-based resources and lay workers to deliver HIV and public health interventions in the face of weak health infrastructure, health care worker shortages and entrenched HIV stigma, is also likely to inform the continued roll out of HIV programmes. 

IAS 2009 was a promising debut for the new operations research track. However, some important programme gaps remain. For example, the critical issues of drug and other medical commodity supply and procurement predictability and forecasting were barely touched on. In addition, the vast majority of data presented in this track related to vertical transmission and ART programmes (including health service integration), with substantially less attention given to prevention interventions beyond circumcision. This is in part due to the research focus of submitted abstracts, as well as the IAS conference’s general focus on biomedical, rather than behavioural prevention interventions.

However, without rigorous cost effectiveness, modelling and programme evaluation studies to help identify the most effective prevention interventions, the evidence base for prevention will continue to lag behind that for care, treatment and support, and questions such as those posed by Bertozzi will continue to be raised about whether the HIV field is making the best use of its hard-won financing.

## Abbreviations

MTCT: Mother to child transmission; VCT: Voluntary counselling and testing; STI: Sexually transmitted infection; IEC: Information, education and communication; IDU: Injecting drug user

## Competing interests

Rodney Kort is an independent consultant contracted by the International AIDS Society for the purpose of drafting this section of the IAS 2009 Impact Report: Summary of Key Research and Implications for Policy and Practice.

## Author’s contributions

RK drafted the initial text, adapted it for publication in a peer-reviewed journal, and approved the manuscript for publication.
